# Unravelling emergency department triage in everyday practice: An ethnographic study of underlying mechanisms across three Danish emergency departments

**DOI:** 10.1186/s12873-026-01612-w

**Published:** 2026-06-30

**Authors:** Cecilie Blomberg Hvid, Michael Dan Arvig, Ove Andersen, Per Nilsen, Christina Østervang, Uswa Nissar Nomani Anjum, Jeanette Wassar Kirk

**Affiliations:** 1 Research Entity for Studies in Clinical aCUte Medicine (RESCUE), Emergency Department, Zealand Hospital, Ingemannsvej 50, Slagelse, Denmark; 2 Department of Clinical Medicine, University of Copenhagen, Blegdamsvej 3B, Copenhagen, Denmark; 3 Department of Regional Health Research, University of Southern Denmark, J.B. Winsløws Vej 19, Odense, Denmark; 4 Department of Clinical Research, Hvidovre University Hospital, Hvidovre, 2650 Denmark; 5 Department of Health, Medicine and Caring Sciences, Linköping University, Linköping, Sweden; 6 Department of Emergency Medicine, Odense University Hospital (OUH), Kløvervænget 25, Odense C, 5000 Denmark; 7 Department of Clinical Research, University of Southern Denmark, Odense, Denmark; 8 Department of Health and Social Context, National Institute of Public Health, University of Southern Denmark, Copenhagen, Denmark

**Keywords:** Emergency department, Emergency ward, triage, Artificial intelligence, Machine learning, Qualitative research, Ethnography, Clinical decision-making, generative mechanisms

## Abstract

**Background:**

Emergency department triage is commonly conceptualised as a standardised classification of patient urgency based on vital signs and presenting symptoms. However, research shows that triage is deeply shaped by the clinical context such as organizational structure, clinical uncertainty and subjective decision-making. This contextual complexity presents a challenge for the implementation of artificial intelligence decision-support in emergency care. While Artificial Intelligence-supported triage has demonstrated promising accuracy in controlled settings, these evaluations capture only a limited part of triage work and rarely account for the underlying mechanisms through which triage is sustained in everyday clinical practice. To address this gap, this study aimed to examine how triage unfolds in real-world emergency care and to understand the underlying generative mechanisms shaping triage practice.

**Methods:**

An ethnographic study was conducted across three Danish emergency departments with different organisational triage configurations. Data consisted of participant observations of triage practice and semi-structured interviews with nurses and physicians. Data were analysed using inductive qualitative content analysis. Findings were interpreted, informed by a critical realistic lens, to understand underlying generative mechanisms shaping triage practice.

**Results:**

Triage was not a standardized classification event but a dynamic, negotiated practice across the acute admission pathway. Two core generative mechanisms were identified: 1) Coherence work sustained continuity, safety, and flow through transitional coordination, gap compensation, situated judgement, and pragmatic prioritisation. 2) Interpretive reasoning enabled healthcare professionals to navigate clinical complexity through situated interpretive filtering and experience-driven judgement. Both mechanisms were activated by managing competing demands between safety, continuity, and flow within fragmented, resource-constrained organisational setups. Both mechanisms drew on tacit and embodied knowledge not routinely documented or available to artificial intelligence systems.

**Conclusions:**

These findings demonstrate that safe and effective triage depends not only on accurate classification but on largely invisible adaptive work embedded in everyday practice under conditions of clinical uncertainty and organisational fragmentation. For artificial intelligence-supported triage to achieve clinical impact, system design must recognise and support the coherence- and interpretive reasoning work through which continuity, safety, and flow are maintained in real-world emergency care.

**Supplementary Information:**

The online version contains supplementary material available at https://doi.org/10.1186/s12873-026-01612-w.

## Background

Emergency departments (EDs) function as the main entry point for acute care within the tax-funded hospital system in Denmark. ED triage is a clinical tool used upon patient arrival to assess urgency and allocate resources [1].

Several different triage systems are used worldwide such as the Emergency Severity Index (ESI), the Manchester Triage System (MTS), and the Danish Emergency Process Triage (DEPT), and while their aim is to ensure consistency in prioritization of patients and staff resources, several challenges remain, such as: over- and under triage, variations of use tied to staff experience, and difficulties assessing complex patient cases [2–4]. These challenges can affect both patient safety and resource utilisation [3]. This points not only to limitations in the design and validity of existing triage tools [5, 6], but also to the complexity of ED environments and the ways tools are implemented [7, 8]. As a result, studies show that healthcare professionals (HCPs) often rely on intuition and tacit knowledge, particularly when dealing with complex or ambiguous situations [9–11]. Other studies demonstrate that triage decisions are further influenced by patient flow, resource availability, teamwork, and departmental routines, making triage a deeply situated practice [7, 12]. Together, this shows that triage operates at the intersection between formal reliability and validity measures and everyday clinical realities, where contextual demands shape how assessments unfolds in practice. This raises the question of how triage is actually interpreted and used in practice. To our knowledge, no studies have examined the underlying mechanisms shaping how triage is interpreted and practiced, a gap that may help explain why longstanding challenges persist in triage. Addressing this gap is also relevant in light of increasing attention toward artificial intelligence (AI)-supported triage tools, which risk being misaligned with clinical practice if the mechanisms shaping everyday triage remain poorly understood.

AI-based triage models have shown promise in improving risk stratification, outcome prediction, and patient prioritization, with several studies demonstrating equal or improved predictive performance compared with traditional triage approaches [13–15].

Despite this, multiple studies reveal a lack of integration of AI into routine care [16–18], leaving a gap in the understanding of the implementation of AI-triage in everyday clinical care.

To optimize the quality of triage and the implementation of AI- triage tools, therefore requires examining the underlying mechanisms that shape how triage unfolds in real-world practice. Underlying mechanisms in line with critical realism (CR), refer to the processes and conditions that are not immediately visible in daily practice but nonetheless shape how HCPs categorize and prioritize patients [19]. These mechanisms may include the judgment strategies HCPs draw upon, the organizational routines and structures that govern workflow and resource allocation, the social and professional norms that influence communication and collaboration, and the material conditions such as spatial layout, and technological tools. Operating across individual, social, and organizational levels, these mechanisms may help explain why certain patterns in triage persist despite standardized systems and formal guidelines, and why triage unfolds differently across contexts [19, 20].

Implementation scientists emphasize that implementation initiatives must be aligned with the context in which practice unfolds [21]. Without this understanding, efforts to improve triage risk being misaligned with everyday clinical practice [12, 21, 22]. This requires attention to the relational, tacit, and processual aspects of triage which can reveal how formal systems, professional judgment, social norms and organizational structures interact and shapes real life triage practice [23]. To our knowledge, no studies have examined the underlying mechanisms through which triage is interpreted and practiced. This represents a gap in understanding why longstanding challenges in triage persist.

Previous qualitative research has advanced understanding of triage as a clinical practice by theorising how triage nurses make decisions in everyday ED work [24]. This provides important insights into individual clinical reasoning in triage contexts. However, triage unfolds across multiple actors, spaces, and organisational boundaries, and less is known about how these broader contextual and organisational processes shape how triage is practiced.

This highlights the need for research that examines triage not only as an individual decision-making activity, but as an ongoing, distributed practice across actors, spaces, and organisational thresholds.

### Aim

This qualitative ethnographic study aims to explore how triage unfold in everyday clinical practice across three Danish emergency departments and to identify the contextual mechanisms shaping its utilisation in clinical practice. The findings are intended to inform improvement initiatives that align with clinical realities.

These insights provide a contextual and conceptual foundation for subsequent research on the design and implementation of AI-supported triage systems grounded in real-world clinical practice.

### Study design and methods

This study is an ethnographic field study using participant observations and semi-structured interviews to capture both how triage is performed and how it is understood by HCPs within the ED. Participant observations (21 days, ≈90 hours) provide access to triage as it unfolds in real time, capturing the practical, often tacit actions and decisions that HCPs make [25, 26]. Semi-structured interviews (mean 14 minutes; range 9–20) complement this by eliciting HCPs´ reflections, rationales, and understandings of their practice – dimensions that may not be visible through observation alone [27]. Together, these methods allow for a thorough understanding of how formal protocols relate to everyday practice.

The study is reported in accordance with the 32-item Consolidated Criteria for Reporting Qualitative Research (COREQ) checklist [28].

### Setting

The study was conducted in three EDs in distinct Danish regions: The Capital Region of Denmark, The Region of Southern Denmark and the Region of Zealand. The EDs varied in size, patient volume, and organizational setup, and included one regional and two university hospitals (Table 1).

**Table 1:** Setting characteristics of the three included Danish EDs

Feature	Site A	Site B	Site C
Organizational Structure	New Joint Emergency Department	Acute Admissions Unit	Joint Emergency Department
Triage model	I-DEPT	I-DEPT	DEPT
Triage Procedure	Pre-hospital + Bedside triage	Pre-hospital +Triage at ambulance entrance/in waiting room + bedside	Pre-hospital phone-based triage
Triage Staffing Model	Nurse led	Nurse led	Nurse led in collaboration with ambulance personnel
ED Bed Capacity	39 treatment spaces and 70 admission beds	13 treatment spaces and 57 admission beds	80 treatment spaces and 47 admission beds
Hospital Bed Capacity	620 beds*	338 beds**	809 beds***

*Based on availabe data from 2024. **based on available data from 2025. ***Based on available data from 2024

*DEPT = Danish Emergency Process Triage (Without individual (I) clinical assessment)*

*I-DEPT = Danish Emergency Process Triage (with individual (I) clinical assessment)*

Danish emergency care is organised according to national principles, but with local variations in how acute units are structured, including joint ED which is an integrated department where multiple specialties are co-located and present at the frontline of acute care and Acute admission units which primarily function as receiving units for initial assessment and short-term treatment and observation before transfer to speciality wards.

Triage was primarily nurse-led across all sites and the formal responsibility for conducting and documenting triage ultimately rested with the ED nurse upon the patient’s arrival to the ED.

The sites were purposefully selected to reflect contextual differences in triage organizational setup, physical layout, and workflow, to explore how local conditions shape ED triage practice.

In Denmark, access to EDs is primarily organised through referral pathways, where most patients are referred either by emergency medical services or by a primary care physician and to a minor degree patients who self-present. Following initial assessment and treatment, patients are either discharged directly or transferred to the appropriate ward, typically within 48 hours.

### Danish triage systems

In Denmark, the Danish Emergency Process Triage (DEPT) (Additional file 1), a five-level symptom-based tool, is used nationally [29]. DEPT assigns patients to five colour-coded urgency levels: life threatening/resuscitation (red), urgent (orange), less urgent (yellow), non-urgent (green), and non-urgent (blue), based on two main descriptors: vital signs and the patient’s presenting complaint, with the most urgent of the two determining the final category of acuity [30]. The system includes scheduled reassessments and supports clinically justified up- and downward adjustments. In some EDs, an extended version of DEPT (I-DEPT) is applied (Additional file 1). I-DEPT combines vital sign measurements with a brief nurse-led clinical assessment, allowing for up-triage by two categories or down-triage by one category. DEPT is inspired by the Swedish Adaptive Process Triage model (ADAPT), and follows the same general sorting principles as the Canadian Triage and Acuity Scale (CTAS) [31].

## Recruitment and participants

### Sampling

Participants were nurses and physicians, included through a purposive sampling strategy to ensure a nuanced range in professional background, triage experience, age, sex and organizational context [32, 33]. Participants were approached face-to-face or by email.

Inclusion criteria required that participants were registered nurses or physicians involved in, and experienced with, triage activities in the ED. Our study aim was relatively broad, which required a sample capturing variation across nurses and physicians. To ensure sample specificity, we included participants with at least six months of ED clinical experience to ensure a minimum level of familiarity and reflective capacity regarding triage practice [34]. Exclusion criteria covered HCPs without clinical responsibility for initial patient assessment, including administrative staff, service staff and nursing or medical students.

The number of participants was not determined in advance but guided by a focus on information richness rather than a pre-fixed sample size. This aligns with the concept of “information power” which emphasizes that the adequacy of a sample depends on the relevance and depth of the data [33]. The interviews produced a rich dataset that was assessed as sufficiently substantial to meet the study aim.

## Participants

A total of 18 participants were included in the interviews, comprising twelve nurses and six physicians, varying in age (28–62 years) and ED experience (1–32 years) (Table 2).

**Table 2:** Interview participant characteristics

Profession	n	Sex (female/male)	Age, median, (range, years)	Acute experience, Median (range, years)
Nurses	12	10/2	42 (31–62)	9 (1.5–32)
Physicians	6	1/5	51 (28–55)	14 [1–18)
**Total**	18	11/7	46 (28–62)	12 (1–32)

### Philosophy of science

The study was informed by a critical realism (CR) paradigm, which guided the formulation of research question, data collection and interpretation. CR is well suited for studying social processes because it assumes that observable actions are shaped by underlying mechanisms such as, routines, norms and organizational conditions, that may not be directly observable but can be studied through empirical inquiry [20, 35].

This perspective was particularly relevant for examining ED triage, where standardised tools coexist with variation in how triage is used in practice. CR enabled the study to move beyond describing observable triage actions to exploring how contextual conditions shape how triage unfolds in everyday clinical practice [35, 36].

This perspective informed the choice of an ethnographic field study consisting of both participant observations and semi-structured interviews.

### Mechanisms

In this study, *underlying mechanisms* are understood in line with CR, as the underlying causal processes that help explain how and why observable patterns of practice occur under specific structural conditions [35]. Mechanisms are not directly observable but are derived from the ways healthcare professionals respond to and adapt within organisational, professional, and material conditions in practice [19, 35].

Mechanisms were understood as operating within particular structural arrangements, such as organisational routines, professional roles, and material environments, and become visible through the adaptive responses healthcare professionals develop within these conditions in everyday practice [19].

## Data collection

### Participant observations

A total of 21 days (≈90 hours) of participant observation of clinical triage practice, each session lasting between 3 and 8 hours, were conducted from September–December 2024 by the first author CBH. CBH a female PhD. Student, has a background as a registered nurse with experience from somatic hospital wards caring for acutely ill patients, providing familiarity with organisational routines and clinical terminology. Without prior ED experience, CBH entered the field as a participant observer, combining familiarity with clinical routines and terminology with an analytic distance to ED practices [26]. Most participants were not previously known to the researcher; however, a small number were familiar through shared organisational affiliations with brief prior professional contact.

Following James Spradley’s ethnographic framework [26], an initial observation guide was developed to capture contextual factors such as actors, places, and activities (Additional file 2), and was iteratively adapted across sites throughout the observations. Observations were carried out in clinical areas where triage was performed, following patient trajectories and related staff activities. Initially, 17 triage nurses were observed as they were assigned to triage duties during that shift. But, as patient trajectories were also followed, additional staff became involved in a more fluid manner, such as physicians, ambulance personnel and ward nurses. All HCPs present during these observations were observed in the clinical environment. The focus was on capturing practices, interactions, and contextual conditions rather than on individual professional characteristics.

CBH began with staff-focused observations to understand organizational structures and routines, before shifting to follow patients through their pathways in the ED. This approach enabled capturing how triage unfolded across internal locations and situations in the ED, through organizational processes, bedside practices and handovers between multiple professionals [37]. Informal questioning was used to clarify situational triage processes and reasoning (e.g. *“what made you choose orange instead of green in this case?”)* with questions posed opportunistically and only when they did not interfere with clinical work. Each day, the researcher was assigned to the triage/flow nurse, beginning in the central triage office and continuing into zones within the ED for bedside triage.

Fieldnotes were written along the way in Danish, expanded shortly after each session, and shared with the research team for reflection and clarification of tacit knowledge [26, 38]. An inductive coding of the fieldnotes was carried out with a focus on text-near meaning units, aiming to identify initial patterns and categories [39, 40]. These preparatory analyses informed four analytical assumptions and the development of the subsequent interview guide (Table 3). This process also supported ongoing reflexive awareness of how the researcher’s professional background and positioning might shape attention and interpretation during both fieldwork and analysis.

**Table 3:** Conceptual framing of the interview guide and analytical questions

Assumptions generated from observations of triage practice	Interview guide (Main questions of the guide to illustrate the connection between assumptions and interview questions)	Additional and follow-up questions exemplified	Analytical questions
Triage is a socially embedded practice Triage practices are shaped and vary according to the context in which they are carried out Triage involves both strengths and limitations Triage is based on the clinical judgement of healthcare professionals	Explain the triage process in your department. Probe: •When and where does triage take place? What is your opinion on triage? Probes: •Describe any strengths •Describe any challenges What is your experience with mis-triage? When you assess a patient during triage, what do you specifically do to make that assessment? Who are your primary collaborators during the triage process? Probes: •Describe the strengths of the collaboration? •Describe the challenges of the collaboration?	How do you prioritize patients arriving with an ambulance and patients arriving through the waiting room? Can you describe a specific recent situation where you used your clinical judgement? Do you experience that you and your collaborators do not agree on the triage decision and how do you mitigate it if it happens?	How do the professionals perceive and experience the triage process in their department? Which strengths do healthcare professionals encounter when they triage patients in the ED? Which challenges do healthcare professionals encounter when they triage patients in the ED? How do different actors involved in triage perceive and describe their collaboration and roles in the decision-making process?

### Interviews

Individual interviews were conducted by CBH between May and June 2025. A total of 18 interviews were conducted using a semi-structured interview guide consisting of five open-ended main questions and several probing questions (Table 3). To strengthen credibility, the interview guide was informed by assumptions identified during observations, including that triage is socially embedded, context-dependent, shaped by both strengths and limitations, and grounded in HCPs professional judgement (Table 3).

The interview guide was piloted using cognitive testing inspired by the think-aloud method on two nurses from one setting and discussed with JWK (RN, PhD, Assoc. Prof.) an experienced qualitative researcher, OA (MD, PhD) professor in emergency medicine and MDA (MD, PhD. in emergency Medicine) an Associate Professor in emergency medicine. The goal was to clarify the relevance of questions and how participants understand and interpret each interview item [41]. Adaptations were made and discussed with JWK to finalize and validate the guide.

The semi-structured interviews had a focused design, aiming to explore a range of aspects of triage practices in the ED and to elaborate on the underlying reasoning and understanding of triage practices from the perspective of HCPs. Due to the busy clinical environment, most interviews were conducted in short breaks between clinical tasks and were therefore concise (mean 14 minutes; range 9–20). However, the data were assessed for richness and depth using the concept of information power, indicating that the sample held sufficient information for analysis [33]. All interviews were digitally recorded and transcribed verbatim by CBH.

### Data analysis

The analysis was structured as a qualitative inductive content analysis (QCA) approach [40, 42], the subsequent development of themes was informed by a critical realist interest in identifying context-sensitive generative mechanisms [19]. Fieldnotes and interview transcripts were managed and coded manually in Microsoft Word 365 (Microsoft Corporation, Redmond, WA, USA). Data from each study site, were analysed separately to explore site-specific features and variations across settings, and were then compared. Coding decisions were continuously discussed between CBH, JWK and OA and disagreements were resolved by consensus. The development from meaning units to categories and themes is illustrated in Additional file 3. The analysis was conducted in four steps:

In the first step, CBH read each transcript several times to gain an overall sense of the dataset while taking notes on initial impressions, reflections, surprises, tensions and broader emerging patterns [40].

Second step included identifying, extracting and condensing meaning units relevant to the study aim using the four analytical questions (Table 3) [1, 40]. 1) How do the professionals perceive and experience the triage process in their department? 2) Which strengths do the professionals encounter when they triage patients in the EDs? 3) Which challenges do the professionals encounter when they triage patients in the EDs? 4) How do different actors involved in triage perceive and describe their collaboration and roles in the decision-making process? In this step, audio files were revisited alongside the transcripts to contextualize meaning units, for example, regarding tone, pauses, and emphasis [40]. The sound files served to support the interpretation of the transcribed material.

In the third step, condensed meaning units were compared based on differences and similarities and sorted into categories, constituting the manifest content [40]. In the fourth step, notes and reflections from interviews were used to contextualize the categories and guide pattern identification, which subsequently informed the formulation of subthemes and overarching themes.

To strengthen credibility, the analytical process was subsequently conducted in dialogue between CBH, JWK, and OA, and finally discussed with MDA.

### Ethics

The study was conducted in accordance with the Declaration of Helsinki [43]. The Danish Regional Committee on Health Research Ethics for Region Zealand, reviewed the protocol and deemed that formal approval was not needed. The project was approved and registered with the Data Protection Officer in Region Zealand (*p*-2024–16716) in compliance with the General Data Protection Regulation and national data protection regulations [44, 45]. Participants received written and oral information about the study aim and provided informed consent. Participation was voluntary, and participants could withdraw at any time without consequences. All participants provided informed consent prior to participation. Personal data were stored securely and anonymised in transcripts, analysis, and reporting.

Ethically responsible conduct was ensured during fieldwork, in line with qualitative fieldwork practices concerning situated presence and researcher–participant interactions [46]. The researcher remained attentive to the clinical workflow and withdrew when staff were called to urgent tasks or seemed uncomfortable with being observed

## Results

The inductive analytical process generated seven categories that were synthesised into six subthemes, and two overarching themes that captured both the perceived and performed dimensions of triage practice among nurses and physicians (Table 4). While the categories reflect the manifest level of the data, the subthemes reveal their latent pattern of the triage process. The analytical process revealed that the categories ‘Physical spaces’ and ‘Roles and functions’ were inherently interrelated and together captured how triage was locally organized. They were therefore synthesized into the subtheme ‘Organizational structures and local variations.

**Table 4:**
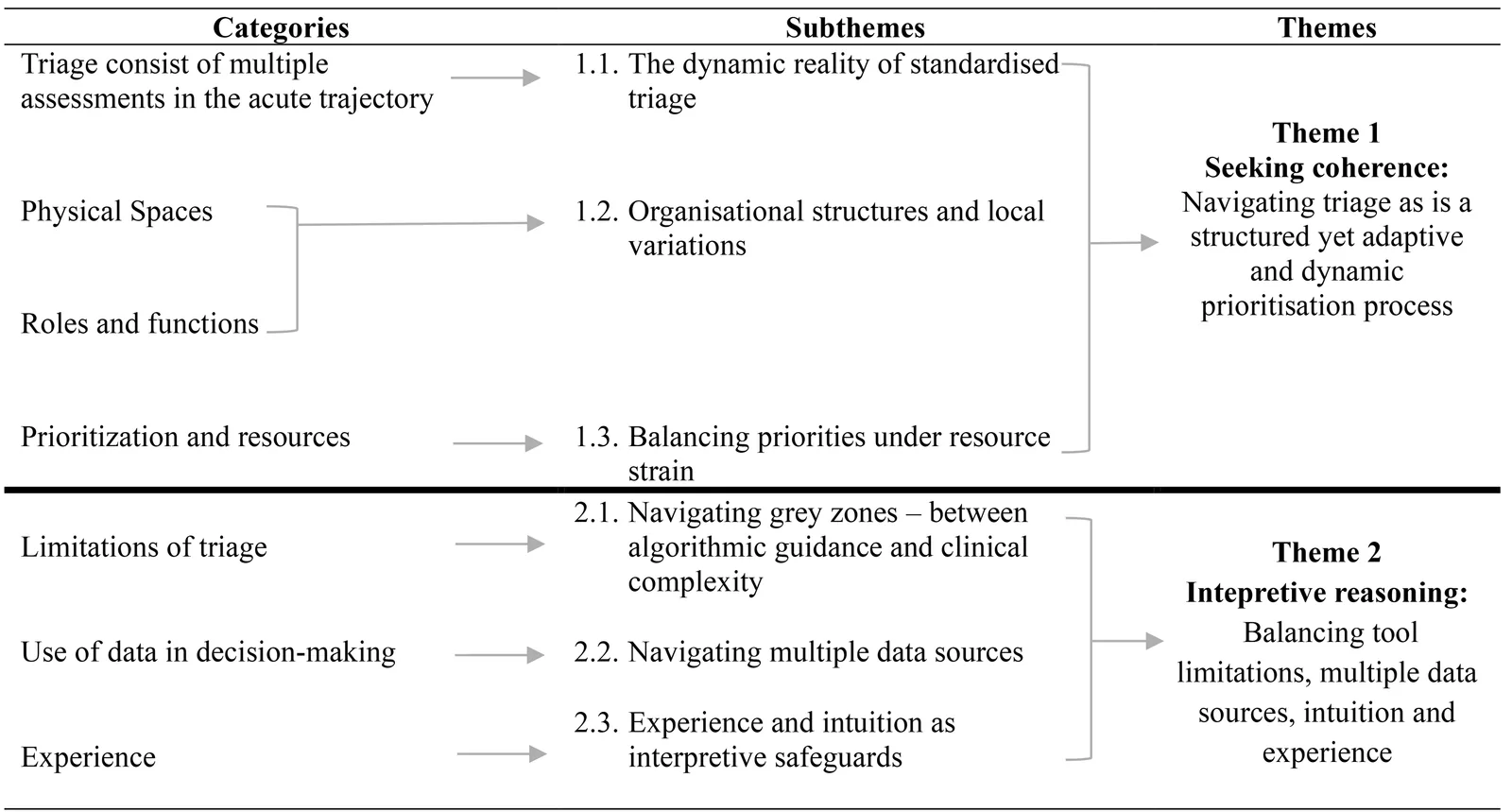
Result overview: from categories to subthemes and themes

Guided by the critical realist analytical lens [35], each theme captured observable patterns and informed an interpretation of potential generative mechanisms that shape how triage unfolds within specific organisational and situational contexts. These mechanisms are analytically elaborated through two themes 1): Seeking Coherence: Navigating triage as a structured yet adaptive and dynamic prioritisation process, and 2) Intepretive Reasoning: Balancing tool limitations, multiple data sources, intuition and experience.

### Theme 1:Seeking coherence: Navigating triage as a structured yet adaptive and dynamic prioritization process

This theme captures how triage unfolds as both a formally structured and contextually adaptive process. While standardized triage tools provide a shared framework, their use is shaped by the continuous movement of patients across organizational boundaries, the structural conditions created by local organizational arrangements, and the pragmatic adjustments required to maintain safety and flow under resource strain.

#### Subtheme 1.1: The dynamic reality of standardised triage

Across all sites, triage was revealed as a continuous and distributed process rather than a single discrete event. Patient priority was assessed, reassessed, and negotiated across multiple encounters, locations, and professional roles as patients moved through the acute care system.

[A patient with headache and chest pain was triaged green prehospital, up-triaged to orange by the ED triage nurse based on a symptom protocol at the triage station, then down-triaged to yellow by the physician after thorough bedside examination. The bedside nurse anticipated this pattern, explaining: “When the physician comes, they typically down-triage to yellow.” The physician explained his reasoning: “The patient is completely ABC-stable.” The patient moved through three urgency assessments within 30 minutes, each reflecting different professional logics and information sources.] (Field note 7, Site B)

Initial prioritization even occurred before the patient’s arrival, when dispatchers assessed urgency and determined ambulance response time:

*In principle, patients are already triaged when they call the emergency dispatcher, where it is essentially an assessment of how quickly the ambulance should be dispatched. That too is a form of triage. Once the ambulance arrives on site, the triage can be revised again, and then we perform a new triage in the ED. *(Interview, Nurse 5)

As patients moved through the system, responsibility and assessment shifted continuously across professional and organisational boundaries:

*You could say that the patient passes through many stages. First, the physician, then the ambulance service, then me, and afterwards the information has to be handed over to another nurse or to the ward upstairs, who will also go through it. But I don’t think it can really be done in any other way. *(Interview, Nurse 7)

Urgency categories were frequently revisited in light of new observations or contextual shifts. Subtle changes in patients’ condition or in how observations were interpreted between HCPs could alter perceived urgency:

*There are situations where I think that patients triaged as yellow should really have been orange. For example, if a patient’s saturation looks fine with oxygen but drops without it, then as the receiving nurse I have a problem. And if the receiving nurse is not fully alert to that, things can go wrong. *(Interview, Nurse 11)

The process was not limited to clinical personnel. Administrative staff were also expected to make initial judgements of urgency:

*When you walk in, you might be spot-triaged by the administrative reception staff, who then judges whether the patient is in severe pain or has special needs. *(Interview, Physician 5)

These accounts illustrate how responsibility and assessment shift continuously across professional and organisational boundaries as information, clinical context, and accountability are redistributed.

#### Subtheme 1.2:Organizational structures and local variations

The triage process unfolded differently across sites, shaped by organisational variations in physical layout, professional roles, and communication structures. While all EDs followed the same national triage protocol (DEPT and I-DEPT), local organizational conditions created distinct contexts in which standardized triage was performed.

### Physical structures: Settings, spaces, and layout

Triage unfolded across several interconnected settings, from the prehospital field to the ED, and through multiple physical spaces within the ED. While all sites followed the same rule that patients triaged red went directly to the trauma room, practices diverged for other triage categories. Across departments, patient allocation followed a shared logic of separating patients by urgency, yet local routines, staffing, and layouts shaped how this logic was performed. In one setting, orange patients were placed in a dedicated unit staffed by experienced nurses. In another, the same category was handled more ad hoc with prioritisation based on resources and available personnel.

The physical layout shaped what could be observed, communicated, and decided. In one site, triage often took place in corridors, waiting areas, or side rooms, each affording different degrees of observation, privacy, and dialogue:

*We do have a triage room, but it is often occupied with patients. What I find difficult is having to triage and identify people in a crowded waiting room, regarding the patient’s privacy and anonymity. So, it limits my ability to get a thorough medical history. Sometimes I just show the patient a note with their identification number and then whisper the referral diagnosis from their general practitioner, to confirm that it is the reason for their visit. *(Interview, Nurse 6)

When space was limited, nurses adapted to secure patient flow:

*If no room is available and the other nurses are busy, then I do the triage myself. It is about getting the patient in as quickly as possible, either into a room, into the corridor, or into a side room. *(Interview, Nurse 1)

### Professional structures: Roles and responsibilities

The organisation of roles and responsibilities revealed different arrangements of professional boundaries and information flow. Across most departments, nurses were the primary professionals responsible for triage, while in one setting this task was shared with prehospital staff:

*In our department, it is primarily the ambulance personnel who carry out triage, pre-hospital. Our ED visitation nurses may ask questions and check whether we should up- or down-triage, but in the end, it is the ambulance personnel who make the triage decision. And it works well. *(Interview, Physician 5)

This collaboration extended triage beyond the ED, with pre-hospital carrying out the physical assessment and triage classification in strict adherence to the triage algorithm while final adjustment was carried out in collaboration with the ED nurse triaging remotely by phone. This increased efficiency and wait times at ED arrival, but reduced the nurses access to sensory and relational information:

*The nurses who triage in our setting often sit in the acute visitation unit and do not have direct contact with the patient. So sometimes there are things that are missed in the triage, which would have been useful to know later in the patient’s pathway. *(Interview, Nurse 11)

In another site, fragmented responsibilities and tasks pulled the triage nurse in multiple directions simultaneously:

[Another patient arrives by emergency transport, suspected hip fracture. The triage nurse goes out to measure vital signs. At the same time, a sense of stress and busyness can be felt across the department. The coordinating nurse comes rushing with an arterial blood gas sample and hands it to the triage nurse: “the patient in room 2 is deteriorating, can you quickly analyze this.” The triage nurse takes the sample and hurries off. Shortly after she returns, saying in frustration: “I will never get to the waiting room”, all while the incoming patient and pre-hospital staff stands in the corridor and waits the nurses direction]

(fieldnote 3, site B)

*When I receive an alarm call with a red triage, I have to go to the trauma room, which means leaving the triage office … It works best if I can stay at my triage station, but much of the time we also have coordinating tasks and care for patients in the fast-track room, and that is a dilemma. *(Interview, Nurse 6)

#### Subtheme 1.3: Balancing priorities under resource strain

Resource constraints made the dynamics of triage particularly visible in everyday work. When workload, staffing, and time limitations intersected with patient intake, professionals relied on situated judgment and informal prioritisation strategies to sustain departmental flow and patient safety.

### Formal timeframes disrupted by limited capacity

Nurses often described frustration when physicians were unable to comply with formal triage timeframes:

*It can be frustrating when a patient who should be seen by a physician within an hour and a half ends up waiting three hours. And a patient triaged as green waits six hours, when they really should wait a maximum of four. *(Interview, Nurse 1)

[During a clinical discussion about down-triaging an orange patient who “seemed fine,” multiple nurses expressed a shared norm of never down-triaging orange patients until physician assessment, due to possible uncertainty about whether abnormal values were habitual. One nurse recounted a recent case where an orange patient experienced cardiac arrest after a three-hour wait, highlighting the safety risks of delays in physician response to triage categories.] (Fieldnote 1, Site A)

While nurses recognised these delays as problematic, they also acknowledged that physicians operated with a broader situational overview, balancing multiple demands simultaneously. From physicians’ perspectives, resource strain not only slowed throughput but also distorted the triage system itself:

*Over-triage is a big problem, especially when there are far too many orange patients. We sometimes have shifts where six or seven orange patients arrive at once, and then it becomes meaningless, because if you only have two physicians and seven orange patients, the whole process just starts over again. *(Interview, Physician 5)

When capacity was exceeded, formal triage categories lost their operational meaning and professionals abandoned strict algorithmic logic in favour of weighing clinical urgency against available resources.

### Informal assessments under resource constraints

Under resource constraints, triage required constant vigilance. In the waiting room, nurses performed ongoing surveillance to ensure patient safety:

*It is necessary to regularly patrol the waiting room to ensure that patients do not deteriorate while waiting. *(Interview, Nurse 4)

Faced with simultaneous arrivals or ambiguous cases, nurses made rapid informal decisions based on partial cues:

*If two or more patients arrive at the same time, you have to judge based on their presenting symptoms and perhaps the handover from the ambulance, or whether they arrive on their own. Then you decide which one to see first. *(Interview, Nurse 2)

In one setting, walk-in patients in the waiting room were systematically deprioritised relative to ambulance arrivals to avoid delaying prehospital staff:

[A steady flow of patients arrives by ambulance. I have lost track of counting. Everyone seems busy. I can see on the screen that three patients are waiting in the waiting room and have been waiting for between 30 and 49 minutes (guidelines say that every patient should be triaged within 15 minutes of arrival). The nurse explains to me that those in the waiting room are unfortunately often deprioritized compared to ambulances, because we can’t have a whole corridor filled with ambulance personnel, they need to move on too. She adds that sometimes patients in the waiting room are actually sicker than those arriving by ambulance. “But that’s just how it is, unfortunately.”] (Fieldnote 2, Site B)

This pattern was not observed or described in the two other settings, suggesting variation in how patient prioritisation was performed depending on local organisational structures and role configurations.

Capacity constraints also necessitated informal bed allocation work. When no designated bed was available for an arriving patient, triage nurses engaged in time-consuming negotiation:

[The nurse has now spent several minutes phoning colleagues across units, and finally trying to persuade a staff member to accept the incoming patient. On the phone the nurse says: “I know it’s not ideal, but there are no other options.” After finishing the call, she turned to me and said that this was simply how things worked: everyone was busy, patients could not be turned away, and someone had to take responsibility for the incoming patient. (Fieldnote 8, Site B)

### Theme 2: Intepretive reasoning – balancing tool limitations, multiple data sources, intuition and experience

This theme captures how HCPs compensate for the limitations of standardised triage tools. Algorithmic parameters frequently fail to capture clinical complexity, creating grey zones where professional judgment becomes essential. The theme reveals how HCP snavigate these dynamics by integrating multiple information sources: ambulance handovers, direct observation, and accumulated clinical experience, to maintain safe prioritisation.

#### Subtheme 2.1: Navigating algorithmic limitations and clinical complexity

HPCs across all settings described persistent tensions between the triage algorithm’s standardised parameters and the complexity of clinical presentations. While the tool provided a shared framework for prioritisation, it frequently failed to capture key clinical cues, particularly in ambiguous or complex patient cases.

### Valuing autonomy while recognising limitations

Nurses generally expressed positive attitudes towards the triage tool, with some valuing its simplicity because it preserved space for professional judgment:

*I would say it is a really good tool. We use I-DEPT, which leaves a great deal with the nurses, a great deal of responsibility. *(Interview, Nurse 5)

Others perceived this simplicity as a limitation compared with earlier, more comprehensive systems. With the newer, simplified systems, newly qualified nurses were seen to lack concrete knowledge of specific rules, for example, that head trauma in patients on anticoagulant treatment should be triaged as orange.

### Triage is a coarse-meshed net

Physicians were particularly critical of the system’s diagnostic limitations:

*The challenge with triage is that it is a coarse-meshed net. This means that some patients slip through who turn out to be more ill than initially assumed. There are certain medical conditions that are simply not captured by triage. *(Interview, Physician 1)

Frequent over-triage in the orange category created recurring frustration and undermined the system’s credibility:

*After running ten times to an orange patient who turned out not to be seriously ill, you start to think: why should I rush this time? *(Interview, Physician 5)

Both physicians and nurses described numerous situations where the algorithm’s parameters failed to capture subtle changes in vital signs, symptoms, or clinical context. At the same time, organisational pressures sometimes created tension between professional judgment and workload management:

*Sometimes, I can sense that the nurses get a bit irritated if I don’t agree to down-triage … They know what that decision triggers … But I hold on to my professional judgement, because in the end, it is my responsibility. *(Interview, Physician 4)

#### Subtheme 2.2: Navigating data, clinical judgment and experience

To compensate for algorithmic limitations, HCPs drew on multiple information sources: ambulance handovers, direct patient observation, electronic records, and accumulated clinical experience, integrating these through judgment and intuition to maintain safe prioritisation.

### Drawing on multiple information sources

Nurses emphasised the importance of direct clinical observation beyond vital parameters:

*Once the patient has arrived, it is our clinical eye combined with the vital signs that guides us … it is about going out to see, touch, feel, and talk with the patient when they arrive. *(Interview, Nurse 8)

This combination of clinical assessment and vital signs involved active judgment about when to accept and when to verify measurements:

[The nurse re-measured the patient’s temperature despite normally relying on prehospital values. She explained that because the temperature measured pre-hospital deviated so much, she wanted to measure it herself. She adds that temperature isn’t part of I-DEPT, but it’s good to know if the patient is hypothermic.] (Fieldnote 2, Site B)

Paramedic handovers contributed valuable contextual knowledge, yet HCPs had to filter assumptions that could distort assessment, particularly regarding socially vulnerable or frequently admitted patients:

*The challenge can sometimes be that paramedics also have an opinion … if there are alcohol bottles everywhere … they conclude the patient is just making a fuss … or that frequent flyers, don’t have anything wrong with them. *(Interview, Nurse 11)

Nurses described actively filtering such information:

*They (the ambulance personnel) deliver what they see, and then I sort through what I can use. *(Interview, Nurse 10)

This interpretive type of filtering was recognised as essential, yet some described where protocol adherence dominated under time pressure:

*As soon as many patients arrive, the pressure increases … under pressure you end up relying only on the numbers and the colour assigned by the system. *(Interview, Nurse 7)

### Experience and intuition as interpretive safeguards

Beyond immediate data sources, accumulated clinical experience functioned as a critical safeguard against the limitations of triage tools. Nurses across all settings emphasised that clinical judgment would ultimately override the algorithm:

*In the end, it is the clinical eye that determines which triage colour they receive. *(Interview, Nurse 10)

Tacit knowledge from earlier, more detailed triage systems continued to inform practice, particularly for less experienced staff:

*Triage is done on the basis of vital parameters, but the old contact-cause cards are still used as support, especially for new nurses*. (Interview, Nurse 4)

HCPs particularly emphasised the importance of experience in detecting risks not reflected in standardised parameters, such as comorbid older patients who appeared stable but were at higher risk:

*Many patients you triage green actually have a mortality rate higher than you would expect, because many of the older patients have comorbidities. But we don’t really take that into account in our context*. (Interview, Nurse 9)

This real-time interpretation, filtering and verification of multiple data sources, and the longer-term mobilisation of accumulated experience and tacit knowledge enabled HCPs to navigate the clinical complexity and, in their view, maintain safe prioritisation.

## Discussion

Across three EDs, we found that triage rarely functioned as a single, time-limited classification event. Instead, urgency was continuously reassessed as patients moved between locations, information was transferred during handovers, and responsibility shifted between professional roles. Across sites, HCPs repeatedly engaged in adaptive coordination and real-time interpretation of patient information to maintain patient safety, continuity, and flow under varying organisational structures and resource conditions. Overall, the findings suggest that triage in everyday practice operates as an ongoing coordination process rather than a discrete one-time assessment. This positions triage as a dynamic and negotiated process [47], shaped by a persistent tension between safeguarding individual patient safety and maintaining system-level flow, reflecting an overarching structural condition of managing competing demands [48].

**Fig. 1:**
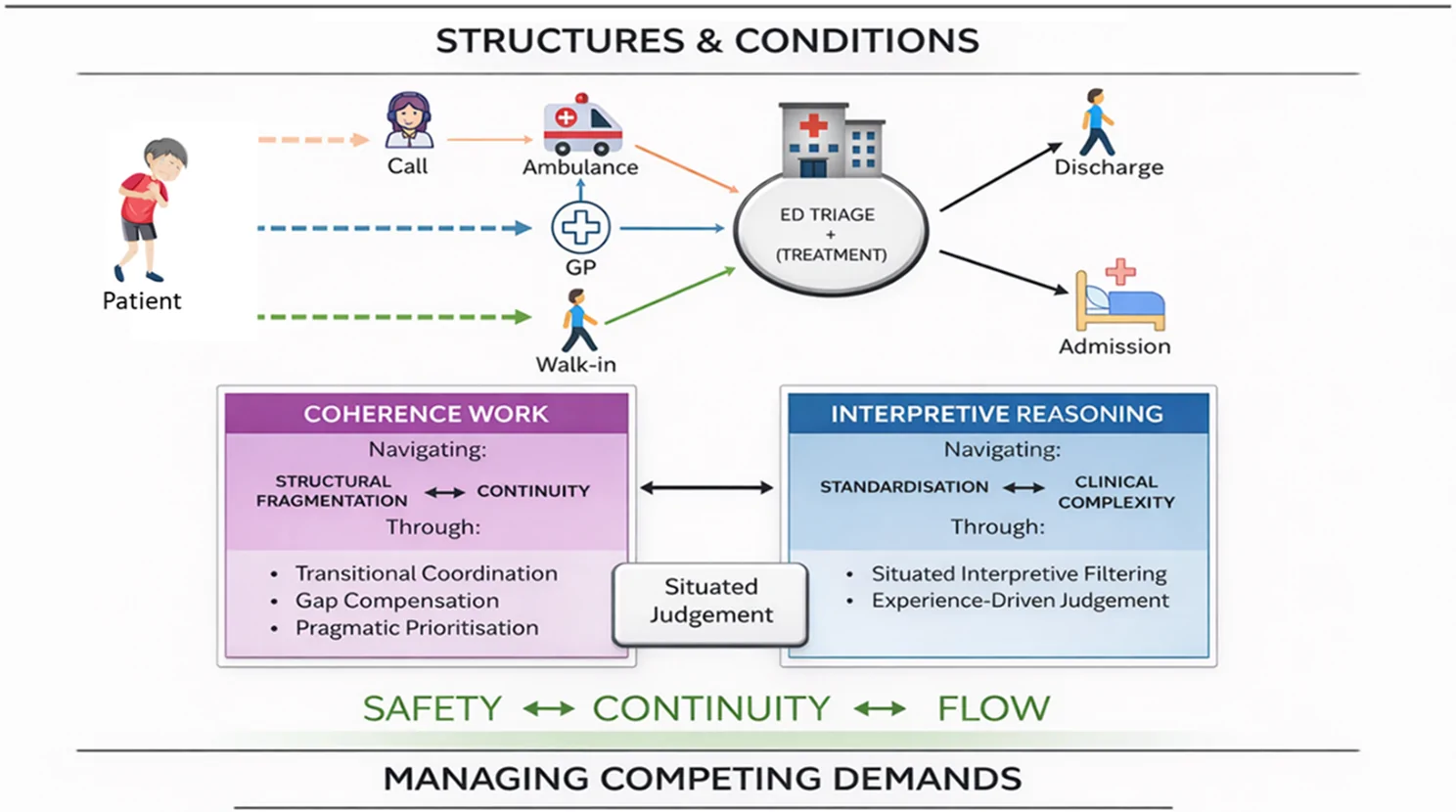
Conceptual model of triage as a dynamic and negotiated practice. The figure illustrates how patients enter the emergency department through three pathways and undergo triage and/or treatment before discharge or admission. Triage is sustained through two core mechanisms: coherence work and interpretive reasoning, which are activated by the structural condition of managing competing demands between safety, continuity, and flow

From a critical realist perspective, these patterns were interpreted as underlying mechanisms that sustain triage under specific structural conditions. Building on the empirically derived themes, the discussion moves to a more explanatory level of analysis [49], identifying two core interrelated mechanisms that enable the HCPs to manage competing demands: coherence work and interpretive reasoning (Fig. 1). These mechanisms are explored in turn in the following sections, followed by an interpretation of their dynamic interplay in shaping triage as an ongoing and negotiated practice.

### Coherence work: Negotiating continuity and safety in everyday emergency care

Coherence work refers to the adaptive, coordinative efforts required to maintain continuity and safety as patients, information, and responsibility move across organizational boundaries. It was activated when organisational fragmentation—arising from incomplete information, role ambiguities, or spatial constraints—created gaps that threatened continuity and safety, requiring HCPs to engage in transitional coordination, gap compensation, situated judgement, and pragmatic prioritisation.

These findings indicate that continuity in emergency care is not maintained solely through protocols or organisational design, but through real-time coordination embedded in everyday clinical practice, where actors, tasks, and temporal demands are continuously aligned rather than the linear application of guidelines [25]. Our findings support existing research emphasizing that triage is shaped by contextual and organisational conditions beyond protocol application, reflecting the complex nature of ED work [24, 25, 50].

This challenges dominant approaches in triage in research, where triage is often framed as a single classification event evaluated through point-in-time accuracy metrics [4, 51, 52]. Similar tendencies are evident in AI-triage studies, where performance is primarily assessed through accuracy outcomes with limited attention to organisational or contextual conditions [13, 53, 54]. While these metrics are valuable for assessing tool performance, our findings suggest that they capture only part of triage work and overlook how urgency is repeatedly reassessed and reinterpreted as patients cross spaces, professional roles, and organisational thresholds.

While coherence work was activated at points where patients or information crossed organisational thresholds, handovers are recognised sources of risk due to discontinuities, delays, and loss of critical cues, affecting both patients and organisational functioning [25]. Whereas successful handovers are often framed as dependent on role clarity and clear communication [25], our findings show that coherence work was activated precisely when such conditions were absent. In those situations, role ambiguities, unclear information flows, and spatial constraints created information gaps and competing tasks, that required transitional coordination and pragmatic prioritisation to sustain continuity, flow, and safety.

Safety in healthcare simultaneously concerns patient trajectory, organisational functioning, and professionals’ capacity to sustain their work [55]. In triage practice, these dimensions were largely maintained through coherence work which in turn, often remains invisible and is primarily recognised when something goes wrong [25]. When safety depends on continuous coherence work, it becomes vulnerable to staffing pressures, system disruptions, and the cumulative cognitive and emotional demands placed on frontline professionals [47, 56]. This may point to a conceptual blind spot in how triage safety is understood and operationalised across research, policy and clinical practice. If triage is treated primarily as a fixed and standardized classification event, the adaptive work sustaining continuity and safety may remain insufficiently recognised and supported in research, tool development, and improvement efforts.

### Interpretive reasoning: Navigating grey zones and clinical uncertainty through professional expertise

Interpretive reasoning refers to the adaptive, interpretive efforts activated when standardized triage parameters fail to capture clinical complexity, creating grey zones in which HCPs must mobilize experiential knowledge and real-time filtering to sustain safe prioritization. It operated through two interrelated dimensions: situated interpretive filtering (real-time weighing and sorting of available information), and experience-driven judgement (mobilising accumulated clinical knowledge and pattern recognition).

Together, these processes enabled HCPs to identify and manage “grey zone” patients, situations in which vital signs, protocol guidance, and patient presentation did not align. This reflects the inherent uncertainty of clinical decision-making, where decisions must be made despite incomplete data and evolving information [57, 58].

There is increasing recognition that clinical expertise is particularly valuable in this grey-scale space, where HCPs must function ambiguity and incomplete knowledge [10, 57, 59]. Our findings extend this understanding by showing how uncertainty is not only cognitively managed, but actively navigated through real-time interpretation of evolving information, contextualisation of numerical indicators, and the mobilisation of experience-based pattern recognition within everyday triage practice. In this sense, experience-driven judgement did not represent a departure from standardisation, but complements situated interpretive filtering by functioning as the human counterpart to numerical indicators in sustaining patient safety.

This aligns with the concept of mindlines, describing tacit, internalised, and socially reinforced clinical knowledge [60]. Although interpretive reasoning appeared as an individual cognitive process in our findings, mindlines theory highlights that such judgement is never formed in isolation [60]. Even when a single nurse made a triage decision alone, the underpinning judgement was shaped by shared observations, informal conversations, local norms, and embedded understandings of what works in the particular ED. This is consistent with emerging empirical work showing that triage decision-making also draws on relational forms of knowledge, including ”know-who” and awareness of expertise or decision authority within the organisation [61]. Collectively, this suggest that interpretive reasoning in triage is a socially embedded practice that reflects and reproduces local ways of recognising risk and urgency [60, 61].

### Interplay across mechanisms: Triage as a dynamic and negotiated practice

Together, the two core generative mechanisms coherence work and interpretive reasoning help explain how triage remains workable under conditions of organisational fragmentation and clinical uncertainty. Although analytically distinguished in this paper, the mechanisms were closely intertwined in everyday practice and often activated simultaneously under shared structural pressures.

Coherence work primarily addressed continuity and fragmentation challenges across organisational thresholds, while interpretive reasoning, addressed clinical uncertainty when standardised triage categories and numerical indicators failed to capture patient complexity. Situated judgement linked these forms of work by enabling HCPs to balance organisational feasibility and clinical significance in real time. Standardised triage tools provided a shared structure, but their effectiveness depended on the continuous interplay between coherence work and interpretive reasoning in everyday practice. Rather than being peripheral to triage practice, they constituted the everyday work through which triage itself remained functional under real-world conditions.

## Implications

The invisibility of coherence work and interpretive reasoning limits opportunities for systematic learning and improvement. If these processes remain unrecognised, decision-support tools, such as triage, risk being designed and implemented in ways that fail to align with everyday clinical practice.

As AI becomes increasingly integrated into emergency care, our findings highlight several considerations. While, to our knowledge, empirical studies examining the organizational and contextual conditions when implementing AI in ED triage are scarce, insights from AI integration in other clinical settings offer relevant perspectives on what is required for AI to function in practice. First, in our study, HCPs relied on clinical cues, including tacit and embodied knowledge, that is not routinely documented. AI systems trained on limited or incomplete inputs risk losing central elements of clinical judgement [62]. Rather than replacing HCPs’ interpretive work in triage, AI may support it by helping to identify patterns, surface uncertainty, compare alternative interpretations, and anticipate possible patient trajectories [63]. Such a role may complement the situated and context-dependent nature of triage. For instance, like illustrated by the implementation of a sepsis prediction system, AI decision-making systems can be designed as part of an interactive workflow that requires HCPs to engage with and verify the AI output before a final decision is made for each patient [64].

Second, AI systems that solely prioritise point-in-time classification may disrupt the coherence work through which continuity and safety are sustained. Similar is seen in a qualitative case study in radiology, where the successful AI integration required active adaptation of existing workflows and redistribution of responsibilities [65]. Likewise, during the implementation of the AI sepsis prediction system, they experienced that alert fatigue among frontline ED staff, could lead to the AI output may failing to reach clinical action, therefore they introduced an existing rapid response team that was repurposed to bridge AI output and clinical ED teams, while also accounting for other clinical needs such as task switching and urgent clinical duties throughout the hospital [64]. Another empirical case study of AI implementation in an emergency department, showed that a decision support system failed to reach adoption due to coordination breakdowns, limited usability for complex multi-complaint patients, and physician distrust following perceived diagnostic errors [66]. This reveal that tools that fail to align with existing coordination and reasoning practices may be overruled in practice, resulting in limited fidelity and minimal impact [67–69].

Future research should thus conceptualize AI -supported triage as a sociotechnical intervention embedded within existing organisational structures rather than as a standalone classifier [70]. Approaches aligned with augmented intelligence that strengthen collective judgement and coordination, rather than replacing them, may better preserve the experiential knowledge through which safety is achieved [71, 72]. Future research should examine how coherence work and interpretive reasoning can be recognised, supported, and strengthened within existing triage practices and during the implementation of new digital tools. Longitudinal sociotechnical studies are needed to understand how professional roles, responsibilities, and reasoning patterns evolve as these mechanisms interact with new technological conditions [70, 73]. Research designs capable of capturing contextual complexity, spatial dynamics, and tacit knowledge will be important for developing interventions and decision-support tools that align with clinical practice.

### Methodological considerations

An ethnographic design was used to explore triage practices in everyday clinical settings, combining observations with semi-structured interviews. Observations provided contextual insight and informed the interview guide and analytical focus; however, fieldnotes were not subjected to full standalone analysis prior to interviews, which may have limited the depth of observational interpretation before the interview phase.

Although the mean interview length was relatively short (14 minutes), this must be understood in light of the ethnographic design. The interviews did not function as standalone data sources but followed approximately 90 hours of participant observation, which constituted the primary empirical foundation.

Including nurses and physicians across three organisational settings with different triage configurations supported variation across professional roles and organisational setups, strengthening transferability to comparable acute care environments.

Data were analysed using inductive QCA [40]. Credibility was strengthened through iterative discussions among researchers with different background, consensus on coding decisions, and a reflexive analytical log [74]. While the close relationship between empirical categories and interpretive subthemes posed a risk of conceptual overlap [74], this was intentional: categories captured empirical manifestations, whereas subthemes and themes articulated the underlying mechanisms.

The three sites were selected to ensure contextual breadth, yet cross-site variation was not treated as an object of comparison but as a way of exploring how similar mechanisms unfolded under different structural conditions. Future explicitly comparative research could examine how particular organiational arrangements activate or constrain specific triage mechanisms. While the study was conducted in a Danish context, the mechanisms identified may resonate with HCPs and researchers in comparable emergency care settings internationally.

The first author’s novice status in qualitative methods may be considered a limitation but was mitigated through regular supervision and collaboration with two experienced qualitative researchers.

Prehospital staff were not included in data collection, limiting insight into how early decision-making that shapes later triage; future studies incorporating prehospital perspectives could strengthen understanding of prioritisation across the full acute care pathway.

## Conclusion

This study demonstrates that triage in everyday emergency care is not a discrete act of classifying urgency, but a dynamic, negotiated practice sustained through generative mechanisms of coherence work and interpretive reasoning. While standardised triage tools provide a necessary structure, their practical effectiveness depends on the often invisible work through which HCPs maintain continuity, safety and flow under conditions of uncertainty and organisational fragmentation.

By conceptualising triage through these mechanisms, the study offers a strengthening existing triage system and developing context-attuned improvement initiatives and technologies, including AI-supported triage, that recognise and support the adaptive work through which safe emergency care is achieved.

## Electronic supplementary material

Below is the link to the electronic supplementary material.

Additional file 1

Additional file 2

Additional file 3

## Data Availability

The datasets generated and/or analysed during the current study are not publicly available due to confidentiality and data protection regulations but are available from the corresponding author on reasonable request and subject to approval by the relevant authorities

## Abbreviation

ED: Emergency Department
AI: Artificial Intelligence
HCPs: Healthcare Professionals
DEPT/I-DEPT: Danish Emergency Process Triage
CR: Critical Realism
QCA: Qualitative Content Analysis
