# Supplementary material for: Unravelling emergency department triage in everyday practice: An ethnographic study of underlying mechanisms across three Danish emergency departments
Source: BMC Emerg Med. 2026 Jun 30;26:254. doi: 10.1186/s12873-026-01612-w (PMC13591733; doi:10.1186/s12873-026-01612-w)
Supplement: Supplementary file 1 [file 12873_2026_1612_MOESM1_ESM.docx]

**Danish Emergency Process Triage (DEPT) Algorithm**

+ Assement based on symptoms. The DEPT manual comprises 53 specific ’symptom cards’, including: chest pain, respiratory symptoms, fever and infection, abdominal pain, and altered level of consciousness etc.

**Translations to English:**

Red= Rescusitation, Orange= Urgent, Yellow = Less Urgent, Green= Non Urgent.

BT_sys_ = Systolic Blood Pressure

KOL= COPD (Chronic Obstructive Pulmonary Disease

O_2_= Oxygen

RF= Respiratory Rate

SpO_2_= SpO₂ measured by pulse oximetry

TP= Temperature

Uden vitalparametre= Without measurent of Vital Parameters

u/ uden= without

m/ med= with

For patients with COPD, SpO_2_ tresholds are reduced with 5%

The figure illustrates the DEPT triage algorithm. Reproduced from *Ugeskrift for Læger* under the Creative Commons CC BY-NC-ND 4.0 license^[1],^^[2]^

**I-DEPT Algorithm**

Figure of The Copenhagen Triage Algorithm (locally referred to as I-DEPT in clinical practice) from: Hasseback m.fl. ^[3]^, licensed under Creative Commons Attribution 4.0 (<http://creativecommons.org/licenses/by/4.0/>).

1. Claus Skriver m.fl., *Triage medfører hurtigere behandling af de mest syge*, nr. 173/40. 3. oktober (2011).
2. “Open Access Creative Commons License”, Ugeskriftet.dk, set 24. februar 2026, https://ugeskriftet.dk/open-access-creative-commons-license.
3. Rasmus Bo Hasselbalch m.fl., “The Copenhagen Triage Algorithm: a randomized controlled trial”, *Scandinavian Journal of Trauma, Resuscitation and Emergency Medicine* 24 (oktober 2016): 123, https://doi.org/10.1186/s13049-016-0312-6.
