# Supplementary material for: Unravelling emergency department triage in everyday practice: An ethnographic study of underlying mechanisms across three Danish emergency departments
Source: BMC Emerg Med. 2026 Jun 30;26:254. doi: 10.1186/s12873-026-01612-w (PMC13591733; doi:10.1186/s12873-026-01612-w)
Supplement: Supplementary file 2 [file 12873_2026_1612_MOESM2_ESM.docx]

# Participant Observation guide

This illustrates the initial observational guide, which were iteratively adapted as observations progressed at each site.
