# Supplementary material for: Unravelling emergency department triage in everyday practice: An ethnographic study of underlying mechanisms across three Danish emergency departments
Source: BMC Emerg Med. 2026 Jun 30;26:254. doi: 10.1186/s12873-026-01612-w (PMC13591733; doi:10.1186/s12873-026-01612-w)
Supplement: Supplementary file 3 [file 12873_2026_1612_MOESM3_ESM.docx]

Analytical process (exemplified): From analytical question to category

From analytical question 🡪 transscribed meaningunit 🡪 condensed meaningunit 🡪 category

| **Analytical Question** | **Meaning units in Danish and translated to English** | **Condensed Meaningunit** | **Category** |
| --- | --- | --- | --- |
| How do the professionals perceive and experience the triage process in their department? | Danish: *I princippet bliver de triageret allerede i det, de ringer 112, kan man sige.*  *Hvor hurtigt skal ambulancen ud og se dem? Det er i princippet også en form for triage. Og så kommer ambulancerne ud på stedet, så kan de lave den triage om. Og så kommer de jo herind, og så laver vi vores egen triage*. (spl 5)  English: *In principle, patients are already triaged when they call the emergency dispatcher, where it is essentially an assessment of how quickly the ambulance should be dispatched. That too is a form of triage. Once the ambulance arrives on site, the triage can be revised again, and then we perform a new triage in the ED.”* (Nurse 5) | Variations of triage (assessments) occur multiple times throughout the patient’s trajectory, performed by different actors on the way toward admission: during the emergency call, in the ambulance, and upon arrival in the ED | triage consist of multiple assessments in the acute admission pathway |
|  | Danish: *Alle patienter bliver jo triageret som indkommende. Der kommer patienterne ind via tre måder hos os. Et, de har været hos egen læge og blevet triageret, eller i hvert fald blevet vurderet i hvertfald, og bliver så sendt ind til os. Så er der selvhenvenderne, og så er der dem, som kommer ind via enten 1813 eller 112. 1813, de har også lavet en vurdering, men det er jo så oftest per telefon, eller ikke oftest, det er per telefon. Og så har vi så 112, som sådan set er patienter, som har ringet 112 patienter.* (Læge 1)  English: *All patients are triaged when they arrive. Patients come in through three pathways here. One: they have seen their GP and been triaged, or at least assessed in some way, and are then sent to us. Then there are the self-presenters. And then there are those who come in via either 1813* or 112**^[1]^*. 1813 has also made an assessment, but that is usually by phone, or not usually, it is by phone. And then we have 112, which are patients who have called 112.* (Physician 1) | Patients are assessed before arrival during the initial referral to the ED: either in person by their GP or by phone via 1813 or 112^1^ |  |

| **Analytical Question** | **Meaning units in Danish and translated to English** | **Condensed Meaning unit** | **Category** |
| --- | --- | --- | --- |
| Which barriers/challenges do healthcare professionals encounter when they triage patients in the ED? | Danish: *Det kommer også an på (ventetid på lægen), hvor travlt lægerne har, og hvor mange akutte, der er kommet før ens egen patient* (Spl 1).  English: *The waiting time for the doctor to see the patients, also depends on how busy the doctors are, and how many acute patients have arrived before your own patient.* (Nurse 1) | Workload determines how long patients wait before being seen by a doctor. | Prioritisation and resources |
|  | Danish: Vi har en triagestue, men den er ofte optaget, så jeg må triagere patienter i venteværelset. Det er svært at sikre privatlivet dér, og jeg bryder mig ikke om at spørge om noget følsomt, når andre kan høre det. Så triagen bliver meget kort, jeg bekræfter bare stille oplysningerne og henvisningsårsagen med patienten. (spl 6)  English: *We do have a triage room, but it is often occupied, so I have to triage patients in the waiting room. It’s difficult to ensure privacy there, and I don’t like asking about sensitive issues when others can overhear. The assessment becomes very brief, I just quietly confirm their details and the referral reason.* (Nurse 6) | The nurse is uncomfortable triaging patients in the waiting room, as it is difficult to protect their anonymity and privacy. Therefore, the triage assessment is often brief and superficial | Physical spaces |
|  | Danish: *Udfordringen kan være, hvis man ikke er så erfaren, kan det godt være nogle skjulte ting, der gør, at jeg måske vil være mere bekymret for en patient, end en, der ikke har så meget erfaring* (spl 2)  English: *The challenge can be that if you are not very experienced, there may be some hidden signs that make me more concerned about a patient than someone with less experience would be* (Nurse 2) | Lack of experience can lead to missing important patient cues | Experience |

| **Analytical Question** | **Meaning units in Danish and translated to English** | **Condensed Meaning unit** | **Category** |
| --- | --- | --- | --- |
| Which enablers/strengths do healthcare professionals encounter when they triage patients in the ED? | Danish: *Hvis man kommer man gående ind, så bliver man spot triageret af en sekretær, der ser om den patient har mange smerter eller har behov for noget særligt eller ej* (læge 5)  English: *When a patient walks in, they are spot-triaged by a secretary, who checks whether the patient is in a lot of pain or has any special needs* (physician 5) | Secretaries spot-triage waitingroom patients for pain and other needs | Triage consist of multiple assessments in the acute trajectory |
|  | Danish: *det er nok en fordel, at man har været sygeplejerske i 20 år. Altså, at man har noget med i rygsækken, så man klinisk kan se, om man skal være bekymret eller ej. Sådan lige nu og her* (Spl7)  English: *It is probably an advantage to have been a nurse for 20 years. You have something in your backpack, so you can clinically see right away whether to be concerned or not, here and now* (Nurse 7) | Experience makes it easier to judge whether to be concerned or not | Experience |
| **Analytical Question** | **Meaning units in Danish and translated to English** | **Condensed Meaning unit** | **Category** |
| How do nurses and doctors involved in triage perceive and describe the collaboration and roles in the decision-making process? | Danish: *lægerne kan jo prioritere anderledes. Det kan jo være, at de har to andre, der også er dårlige, og så bliver de måske prioriteret over det… Jeg har jo kun mit eget lille overblik, hvis jeg står i en gruppe. Så går jeg ud fra, at de har det samlede overblik og vurderer, hvilke patienter, der skal ses* (Nurse 2)  English: *The physicians may prioritize differently. It could be that they have two other patients who are also in a bad condition, and then those are prioritized instead.… I only have my own limited overview when I am standing in one group of patients, so I assume they have the broader overview and decide which patients should be seen.”* (Nurse 2) | Physicians may prioritise differently because doctors and nurses have different patients and responsibilities. | Prioritisation and ressources |

# Analytical process (exemplified): From meaning unit to sub-theme

From meaning unit 🡪 category 🡪 subtheme and theme

| **Theme 1:** Seeking Coherence: Triage as a structured yet adaptive and dynamic prioritisation process | | |
| --- | --- | --- |
| **Meaningunits (exemplified)** | **Category** | **Sub-theme** |
| Triage is performed upon arrival, except for patients referred through 1813, who are in a way already pre-triaged (Nurse 5)  There are many steps and variations of triage the patient must go through before admission: the doctor, the ambulance staff, the triage nurse, and the receiving nurse. And patients are triaged in different locations depending on their mode of arrival: at the ambulance entrance or in the waiting room by the triage nurse, and bedside by the receiving nurse (Nurse 10)  The patients are triaged in multiple places and times: Dedicated triage rooms, ambulance or trauma rooms. (Phycisian 6) | Triage consist of multiple assessments in the acute trajectory | The dynamic reality of standardised triage |
| It is a problem that patients are triaged in the waiting room because of anonymity concerns (Nurse 10)  Variation in practice and transitions across departments can lead to important clinical changes being overlooked during handover (Nurse 11) | Physical Spaces | Organisational structures and local variations |
| The triage nurse often must leave the triage area for other tasks, such as preparing trauma room notifications, which creates practical challenges in moving between the ambulance entrance and the waiting room. The multiple simultaneous responsibilities, including coordination and patient care, make it difficult to maintain continuous triage and create an organisational dilemma (Nurse 6)  It is problematic that one nurse has to cover two critical functions for large parts of the day, as this limits the time available for triage (Nurse 8)  Prehospital ambulance staff perform the triage assessment, and ED nurses only adjust if needed (Physician 5) | Roles and functions | Organisational structures and local variations |
| When physicians do not adhere to the triage-defined timeframes for assessment, it creates frustration among the nurses (Nurse 1)  When several patients arrive at the same time, decisions about who to triage first are based on symptoms and the handover information (Nurse 2)  When the ambulance workload is high, patients in the waiting area are deprioritised, which can significantly delay their triage (nurse 6)  Since there is no guideline for who to triage first, an informal prioritization is made by secretaries and nurses who visually scans the waiting room while also considering any suspected diagnoses the patients have been reported with (Nurse 12) | Prioritization and resources | Balancing priorities under resource strain |
| **Theme 2:** Clinical Judgement: balancing tool limitations, multiple data sources, intuition and experience | | |
| **Meaning units (exemplified)** | **Category** | **Sub-theme** |
| A typical cardiology patient may present with chest pain but have normal vital signs in the beginning of the illness course (Nurse 3)  Triage functions like a coarse-meshed filter and may overlook serious conditions such as hypokalaemia and kidney failure (Physician 1)  COPD patients with low oxygen saturation and mild tachycardia after treatment often trigger a red alarm in the system, even though they appear clinically stable. You are only allowed to downgrade one category, so they end up as orange, but clinically they would often be assessed as yellow (nurse 10)  A considerable proportion of patients assigned orange priority do not, in practice, present with an acute clinical need (physician 3)  Some patients presents with normal vital signs and are triaged green or yellow on the algorithm, but are in fact very sick (physician 6, Site C) | Limitations of triage | Navigating greyzones – between algorithmic guidance and clinical complexity |
| Clinical judgement goes beyond vital signs: observing appearance, breathing, consciousness, and listening to the patient and relatives. Age, comorbidities, life situation, and deviations from the patient’s usual condition are all considered, as well as a ‘gut feeling’ when something seems wrong (nurse 1)  The nurse’s clinical assessment is based on the patient’s history and on observations of signs such as sweating, unequal pupils, cyanotic legs, and other physical indicators (Nurse 9)  If the ambulance staff reports a possible diagnosis, e.g., ‘suspected pneumonia,’ the clinical focus may become anchored to it, even though the underlying cause might be different. It requires attentiveness to independently assess the symptom presentation instead of relying on the preliminary diagnosis (Nurse 10)  The nurse continuously monitors reports and vital values in the pre-hospital electronic patient record to form an initial impression of the patient’s condition, while remaining aware that the actual situation may differ when the patient arrives (Nurse 7) | Use of data in decision-making | Navigating multiple sources of data |
| Triage is based on early warning score, but the old more comprehensive symptom cards are still used as a support. However this knowledge is not necessarily visible or known to new nurses (Nurse 4)  The physician finds that the triage algorithm tends to over-triage, so a visual assessment of whether the patient appears ill is used; however, this can be difficult for less experienced doctors and nurses who have limited clinical experience (Physician 3)  The nurse initially experienced the triage model as rigid and believed one should adhere strictly to it, but also relies on clinical judgement and intuition to deviate from the system when deemed necessary (Nurse 6)  Since the triage tool aren’t that accurate, it can be a challenge for new nurses and physicians (physician 5) | Experience | Experience and intuition as interpretive safeguards |

1. 1813 Is a regional urgent care telephone service providing medical advice and referral outside GP opening hours

   112 is the national emergency number (equivalent to 911 in the US / 999 in the UK)
